# Effects of 16 Genetic Variants on Fasting Glucose and Type 2 Diabetes in South Asians: *ADCY5* and *GLIS3* Variants May Predispose to Type 2 Diabetes

**DOI:** 10.1371/journal.pone.0024710

**Published:** 2011-09-20

**Authors:** Simon D. Rees, M. Zafar I. Hydrie, J. Paul O'Hare, Sudhesh Kumar, A. Samad Shera, Abdul Basit, Anthony H. Barnett, M. Ann Kelly

**Affiliations:** 1 College of Medical and Dental Sciences, University of Birmingham, Birmingham, United Kingdom; 2 Baqai Institute of Diabetology and Endocrinology, Karachi, Pakistan; 3 Warwick Medical School, University of Warwick, Coventry, United Kingdom; 4 Diabetic Association of Pakistan, Karachi, Pakistan; 5 BioMedical Research Centre, Heart of England NHS Foundation Trust, Birmingham, United Kingdom; University of Bristol, United Kingdom

## Abstract

**Background:**

The Meta-Analysis of Glucose and Insulin related traits Consortium (MAGIC) recently identified 16 loci robustly associated with fasting glucose, some of which were also associated with type 2 diabetes. The purpose of our study was to explore the role of these variants in South Asian populations of Punjabi ancestry, originating predominantly from the District of Mirpur, Pakistan.

**Methodology/Principal Findings:**

Sixteen single nucleotide polymorphisms (SNPs) were genotyped in 1678 subjects with type 2 diabetes and 1584 normoglycaemic controls from two Punjabi populations; one resident in the UK and one indigenous to the District of Mirpur. In the normoglycaemic controls investigated for fasting glucose associations, 12 of 16 SNPs displayed β values with the same direction of effect as that seen in European studies, although only the *SLC30A8* rs11558471 SNP was nominally associated with fasting glucose (β = 0.063 [95% CI: 0.013, 0.113] *p* = 0.015). Of interest, the *MTNR1B* rs10830963 SNP displayed a negative β value for fasting glucose in our study; this effect size was significantly lower than that seen in Europeans (*p* = 1.29×10^−4^). In addition to previously reported type 2 diabetes risk variants in *TCF7L2* and *SLC30A8*, SNPs in *ADCY5* (rs11708067) and *GLIS3* (rs7034200) displayed evidence for association with type 2 diabetes, with odds ratios of 1.23 (95% CI: 1.09, 1.39; *p* = 9.1×10^−4^) and 1.16 (95% CI: 1.05, 1.29; *p* = 3.49×10^−3^) respectively.

**Conclusions/Significance:**

Although only the *SLC30A8* rs11558471 SNP was nominally associated with fasting glucose in our study, the finding that 12 out of 16 SNPs displayed a direction of effect consistent with European studies suggests that a number of these variants may contribute to fasting glucose variation in individuals of South Asian ancestry. We also provide evidence for the first time in South Asians that alleles of SNPs in *GLIS3* and *ADCY5* may confer risk of type 2 diabetes.

## Introduction

Type 2 diabetes, a disease that is 4- to 6-fold more common in South Asian individuals than Europeans, is characterised by impaired glucose homeostasis resulting from a combination of beta cell dysfunction and insulin resistance. This inability to adequately regulate blood glucose levels is also linked to both micro- and macro-vascular complications. Recently, the Meta-Analysis of Glucose and Insulin related traits Consortium (MAGIC) identified 16 genetic variants robustly associated with fasting glucose in non-diabetic populations of European origin [1]. Although a number of these single nucleotide polymorphisms (SNPs) were also associated with type 2 diabetes, several were not, suggesting that some variants may be associated with a ‘physiological’ variation in glucose levels without influencing ‘pathological’ variation and type 2 diabetes risk [1]. As with other genetic associations, replication of these findings in datasets of different ethnic origin is an important step in helping to fine-map the aetiological variants at these loci. Our aim was to investigate the effect of these 16 SNPs on fasting glucose levels and type 2 diabetes in South Asian populations of Punjabi ancestry.

## Materials and Methods

### Ethics statement

Informed written consent was obtained from all study participants and the studies were approved by the Birmingham East, North and Solihull Research Ethics Committee (UKADS participants) and the Baqai Institute of Diabetology and Endocrinology Institutional Review Board (DGP participants). UKADS registered clinical trial number; ISRCTN38297969.

### Study design

Participants in the investigation (Table 1) were from two studies of South Asian individuals; the United Kingdom Asian Diabetes Study (UKADS; 857 participants with type 2 diabetes, 417 without) [2], [3] and the Diabetes Genetics in Pakistan study (DGP; 821 participants with type 2 diabetes, 1167 without) [3]. Participants were all of Punjabi ancestry, confirmed over three generations, and originated predominantly from the District of Mirpur, Pakistan. Diagnosis of type 2 diabetes was established using World Health Organisation criteria [4]. Normoglycaemic control subjects were recruited from the same geographical region as subjects with type 2 diabetes. In the UKADS control group normal glucose tolerance was defined in the majority of participants as random blood glucose <7 mmol/l. In a small number of UKADS controls (n = 22), normoglycaemia was defined as fasting plasma glucose <6.1 mmol/l and 2 hr plasma glucose <7.8 mmol/l on a 75 g OGTT; due to small sample size these data were not included in any fasting glucose analyses. Normal glucose tolerance in the DGP control group was defined as fasting whole blood glucose ≤5.6 mmol/l. Genomic DNA was extracted either from venous blood using the Nucleon® protocol (Nucleon Biosciences, Coatbridge, UK) (UKADS) or from saliva using the Oragene® DNA sample collection kit and extraction protocol (DNA Genotek Inc., Ontario, Canada) (DGP). The clinical details of individuals from the two study populations are shown in Table 1.

**Table 1:** Demographic and health characteristics of study participants in the two populations.

	UKADS		DGP	
	Controls	T2D cases	Controls	T2D cases
n (male/female)	217/200	388/469	617/550	430/391
Age (years)	54.9 (11.7)	56.9 (12.0)	56.3 (10.8)	54.6 (11.7)
Fasting plasma glucose (mmol/l)	−	−	5.5 (0.6)	−
Random blood glucose (mmol/l)	5.3 (0.9)	−	−	−
HbA_1c_ (%)	−	8.3 (1.9)	−	9.6 (3.2)
BMI (kg/m^2^)	28.0 (4.9)	28.6 (4.6)	24.3 (5.0)	26.1 (4.7)

All values except (n) are means (SD). T2D = type 2 diabetes. Within the UKADS control group BMI data were only available for 256 subjects.

### Genotyping

All subjects (UKADS, n = 1274; DGP, n = 1988; total, n = 3262) were genotyped for the 16 SNPs using the KASPar method (KBioscience, Hoddesdon, UK). Genotyping success rates were >96% for each SNP. Approximately 10% of samples were genotyped as blind duplicates resulting in an error rate of <1% for each SNP. Genotype counts in the two study populations are shown in Table S1.

### Statistical analyses

Deviation from Hardy-Weinberg equilibrium (HWE) in the non-diabetic groups was tested using an exact test implemented in Haploview [5]. None of the SNPs deviated significantly from HWE after adjustment for multiple testing. Linear regression was used to test for association between SNPs and fasting glucose levels in the DGP control group only, adjusting for age, sex and BMI. Four DGP control participants had missing BMI data; the sample size for the fasting glucose analysis was therefore 1163. Fasting plasma glucose estimates were calculated from fasting whole blood measurements using a conversion factor of 1.15. Association between SNPs and type 2 diabetes was tested in the UKADS and DGP study populations separately, using logistic regression, adjusting for age and sex. Inverse variance weighted meta-analysis, implemented in Metan, was used to combine ORs from the UKADS and DGP study populations. Only 256 UKADS controls had available BMI data. As BMI made little difference to the type 2 diabetes association (combined UKADS/DGP OR difference ≤0.015 for all SNPs), to maintain sample size and power the main results are not adjusted for BMI. Heterogeneity of ORs (between UKADS and DGP study populations, and between the combined UKADS/DGP cohort and previously published studies) was assessed using Cochran's Q statistics. None of the studied SNPs displayed significant heterogeneity of ORs between the UKADS and DGP study populations after correcting for multiple testing (Table 2). For all single-locus analyses in this study, two levels of statistical significance are referred to; nominal significance (*p*<0.05) and study-wide significance (*p*<3.13×10^−3^, corrected for 16 independent tests). Genetic risk scores (GRSs) were constructed to investigate the additive effect of multiple SNPs on fasting glucose levels and type 2 diabetes. For all GRS analyses, only those individuals successfully genotyped at ≥12 SNPs (n_max_ = 3231) were included. Firstly an allele count GRS (acGRS) was constructed. For each individual, the average number of risk alleles per successfully genotyped SNP (total number of observed risk alleles/number of successfully genotyped SNPs) was multiplied by the total number of SNPs included in the GRS. This produced a GRS approximating a simple risk allele count where all SNPs were successfully genotyped. Two weighted GRSs (wGRS) were also produced, one for the fasting glucose analyses and one for the type 2 diabetes analyses. Risk alleles of each SNP were weighted by a SNP-specific β-value, obtained from the MAGIC study [1], and the weighted allele count was divided by the mean MAGIC-derived β-value for the successfully genotyped SNPs. GRSs for the type 2 diabetes analyses were also estimated without the *TCF7L2* and *SLC30A8* variants, as these SNPs have previously displayed association with the disease in the UKADS and DGP study populations [3], [6]. All statistical analyses were performed using Stata IC Version 10.1 (Stata Corporation, College Station, TX, USA). Power was calculated using Genetic Power Calculator (http://ibgwww.colorado.edu/~pshaun/gpc/) [7] and Quanto version 1.2 (http://hydra.usc.edu/gxe) [8], assuming a significance level (α) of 0.05.

**Table 2:** Association of SNPs with type 2 diabetes in the UKADS and DGP study populations.

			UKADS			DGP			UKADS/DGP			
Nearest gene	SNP	Allele(Risk/other)	RAF	OR (95% CI)	*p*	RAF	OR (95% CI)	*p*	RAF	OR (95% CI)	*p*	Heterogeneity*p*
*MTNR1B*	rs10830963	G/C	0.42	0.93 (0.79, 1.10)	0.403	0.39	0.99 (0.88, 1.13)	0.92	0.40	0.97 (0.88, 1.07)	0.558	0.544
*ADRA2A*	rs10885122	G/T	0.79	0.94 (0.76, 1.15)	0.525	0.75	1.13 (0.98, 1.32)	0.10	0.76	1.06 (0.94, 1.20)	0.339	0.138
*C2CD4B*	rs11071657	A/G	0.70	0.93 (0.77, 1.12)	0.458	0.68	0.92 (0.80, 1.05)	0.21	0.68	0.92 (0.83, 1.03)	0.147	0.899
*SLC30A8*	rs11558471	C/T	0.71	1.11 (0.93, 1.34)	0.242	0.74	1.14 (0.99, 1.33)	0.07	0.73	1.13 (1.01, 1.27)	0.034	0.827
*CRY2*	rs11605924	A/C	0.48	1.14 (0.96, 1.35)	0.143	0.49	0.94 (0.82, 1.06)	0.30	0.49	1.00 (0.91, 1.11)	0.974	0.072
*ADCY5*	rs11708067	A/G	0.74	1.26 (1.04, 1.54)	0.019	0.77	1.21 (1.03, 1.41)	0.02	0.76	1.23 (1.09, 1.39)	0.001	0.715
*SLC2A2*	rs11920090	T/A	0.85	1.13 (0.89, 1.43)	0.325	0.85	0.94 (0.78, 1.12)	0.47	0.85	1.00 (0.87, 1.15)	0.997	0.222
*FADS1*	rs174550	T/C	0.81	1.02 (0.82, 1.26)	0.869	0.81	1.19 (1.00, 1.41)	0.05	0.81	1.12 (0.98, 1.28)	0.095	0.264
*GCK*	rs1799884	A/G	0.16	0.79 (0.62, 1.00)	0.047	0.15	1.05 (0.88, 1.25)	0.58	0.15	0.95 (0.82, 1.09)	0.461	0.055
*DGK/TMEM195*	rs2191349	T/G	0.62	0.93 (0.78, 1.11)	0.418	0.60	1.16 (1.02, 1.32)	0.02	0.60	1.07 (0.97, 1.19)	0.179	0.044
*PROX1*	rs340874	C/T	0.59	1.00 (0.84, 1.18)	0.956	0.59	0.97 (0.85, 1.10)	0.60	0.59	0.98 (0.88, 1.08)	0.656	0.787
*G6PC2*	rs560887	C/T	0.82	1.22 (0.97, 1.53)	0.084	0.84	0.89 (0.75, 1.06)	0.19	0.84	1.00 (0.87, 1.14)	0.993	0.030
*GLIS3*	rs7034200	A/C	0.48	1.14 (0.96, 1.35)	0.133	0.48	1.18 (1.04, 1.34)	0.01	0.48	1.16 (1.05, 1.29)	0.003	0.748
*GCKR*	rs780094	C/T	0.74	0.87 (0.72, 1.05)	0.139	0.72	1.07 (0.93, 1.23)	0.35	0.73	0.99 (0.89, 1.11)	0.883	0.081
*TCF7L2*	rs7903146	T/C	0.31	1.25 (1.05, 1.49)	0.014	0.32	1.22 (1.07, 1.40)	0.00	0.32	1.23 (1.11, 1.37)	0.000	0.870
*MADD*	rs7944584	A/T	0.77	1.01 (0.83, 1.23)	0.893	0.78	1.12 (0.96, 1.31)	0.15	0.78	1.08 (0.95, 1.22)	0.230	0.436

UKADS = UK Asian Diabetes Study; DGP = Diabetes Genetics in Pakistan; Risk allele is the fasting glucose raising allele reported in Dupuis et al [1]; RAF = risk allele frequency, calculated using the normoglycaemic control groups.

The *GCK* rs1799884 SNP was used as a proxy for the rs4607517 variant reported in Dupuis et al [1] (r^2^ = 1.0 in CEU HapMap samples).

## Results

### Associations with fasting glucose

**Figure 1:**
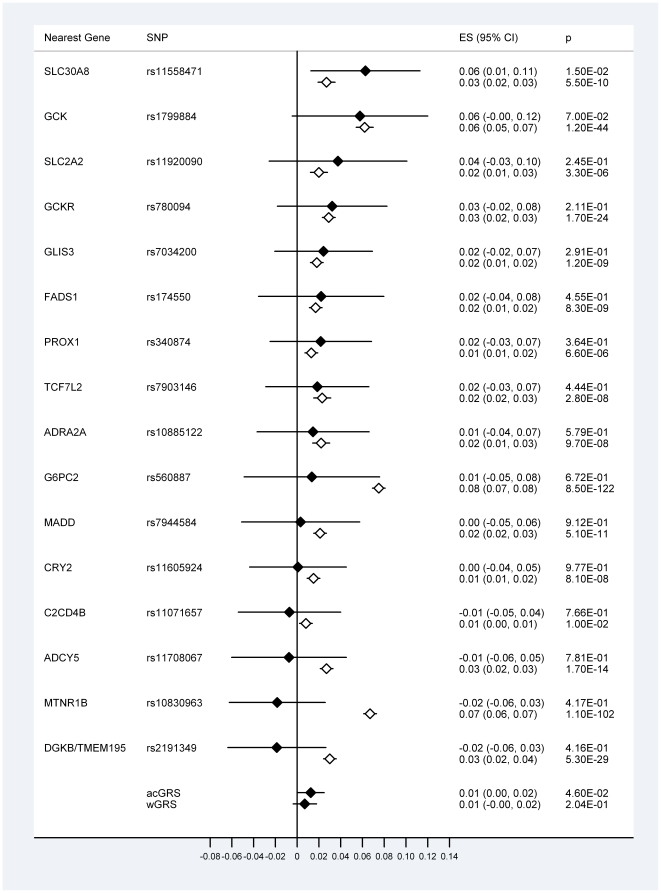
Association of 16 SNPs with fasting glucose in South Asians and Europeans. South Asians from the current study (filled diamonds, n = 1163) and Europeans from the MAGIC [1] study (unfilled diamonds, n≤76,558). acGRS = allele count GRS, wGRS = weighted GRS. ES = effect size, the per-risk allele change in fasting glucose (mmol/l). The *GCK* rs1799884 SNP was used as a proxy for the rs4607517 variant reported in Dupuis et al [1] (r^2^ = 1.0 in CEU HapMap samples).

Of the 16 SNPs studied, 12 displayed β-values with the same direction of effect as that seen in Europeans, and the *SLC30A8* rs11558471 variant displayed a nominally significant association with fasting glucose levels (β = 0.063 [95% CI: 0.013, 0.113] *p* = 0.015) (Figure 1). No evidence for heterogeneity of effect sizes between our study and a previous study of Europeans was apparent for 15 of the studied variants. In contrast, the *MTNR1B* rs10830963 effect size was markedly lower in the current study compared with the Europeans from the MAGIC study [1] and this heterogeneity achieved study-wide significance (*p* = 1.29×10^−4^). The acGRS showed a stronger association with fasting glucose than did the wGRS (β = 0.013 [95% CI: 0.000, 0.025] *p* = 0.046 and β = 0.007 [95% CI: −0.004, 0.018] *p* = 0.204 respectively) (Figure 1).

### Associations with type 2 diabetes

**Figure 2:**
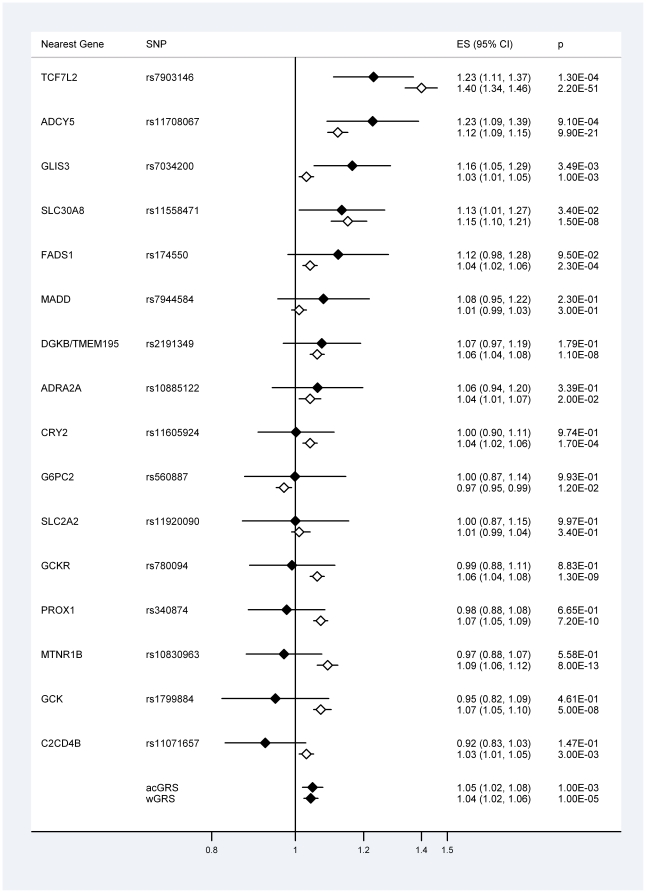
Association of 16 SNPs with type 2 diabetes in South Asians and Europeans. South Asians from the current study (filled diamonds, n = 3262) and Europeans from the MAGIC [1] study (unfilled diamonds, n≤127,667). acGRS = allele count GRS, wGRS = weighted GRS. ES = effect size, the per-risk allele odds ratio. The *GCK* rs1799884 SNP was used as a proxy for the rs4607517 variant reported in Dupuis et al [1] (r^2^ = 1.0 in CEU HapMap samples).

None of the 16 studied variants displayed strong evidence for heterogeneity of effect on type 2 diabetes risk between the current study and the MAGIC study. In addition to the *TCF7L2* and *SLC30A8* variants, which have previously demonstrated association with type 2 diabetes in the UKADS/DGP study populations [3], [6], alleles of the *ADCY5* rs11708067 (OR = 1.23 [95% CI: 1.09, 1.39] *p* = 9.10×10^−4^) and *GLIS3* rs7034200 (OR = 1.16 [95% CI: 1.05, 1.29] *p* = 3.49×10^−3^) SNPs conferred risk of the disease in this study (Figure 2). The strength of these associations reached study-wide significance for the *ADCY5* variant; the *GLIS3* SNP failed to reach this threshold by a narrow margin. Both risk score measures were associated with type 2 diabetes, with the wGRS displaying a stronger association (OR = 1.04 [95% CI: 1.02, 1.06] *p* = 1.00×10^−5^) than the acGRS (OR = 1.05 [95% CI: 1.02, 1.08] *p* = 0.001) (Figure 2). The strength of association of both risk scores was greatly attenuated by the removal of the previously associated *TCF7L2* and *SLC30A8* variants (wGRS; OR = 1.01 [95% CI: 0.990, 1.04] *p* = 0.261, acGRS; OR = 1.03 [95% CI: 1.00, 1.06] *p* = 0.071).

## Discussion

In this study we investigated the effects of 16 SNPs on fasting glucose levels and type 2 diabetes risk in two South Asian populations of Punjabi ancestry. Only the *SLC30A8* rs11558471 variant was nominally associated with fasting glucose levels. Twelve of the 16 SNPs displayed positive β-values, however, suggesting that a number of these variants may be true determinants of fasting glucose levels in our study populations (Figure 1), even though their effects were too small to be accurately detected in our modestly-sized cohort. Comparing the effect sizes observed in this study to those reported in Europeans highlights some potential differences. Three of the six variants most strongly associated with fasting glucose in the MAGIC study [1](*MTNR1B* rs10830963, *DGKB/TMEM195* rs2191349 and *ADCY5* rs11708067) have negative β-values in the current study (Figure 1), and this disparity reached statistical significance for the *MTNR1B* variant. The observed differences in effect size are probably the reason that the acGRS was nominally associated with fasting glucose levels, but the wGRS (weighted using European-derived β-values) was not.

It is of interest to note that variants within or near *GCK*, *GCKR*, *G6PC2* and *MTNR1B* have been shown to be associated with fasting glucose levels in Indian Asians, with similar effect sizes to those seen in Europeans [9]. The *GCK* and *GCKR* SNPs studied in Indian Asians (rs4607519 and rs1260326 respectively) are in strong linkage disequilibrium (LD) with the SNPs genotyped in this study (r^2^≥0.96 in GIH HapMap [10] samples; Gujerati Indians in Houston, Texas) and the *G6PC2* rs560887 SNP was genotyped in both studies. The fasting glucose association results we present for *GCK* and *GCKR* are very similar to those demonstrated in both the Europeans from the MAGIC [1] study (Figure 1) and Indian Asians [9]. Although our estimate of effect size for the *G6PC2* rs560887 variant is low (Figure 1), it is not statistically different from that seen in Europeans. In contrast, the *MTNR1B* rs10830963 variant displayed an effect size lower than that seen in Europeans, at study-wide significance. The *MTNR1B* variant reported as being associated with fasting glucose in Indian Asians [9] (rs2166706) is only in moderate LD (r^2^ = 0.45) with the variant (rs10830963) genotyped in our South Asian cohort and reported as the sentinel *MTNR1B* SNP in the MAGIC study [1]; if different LD patterns result in rs2166706 being in tighter LD with the aetiological variant than rs10830963 in South Asians, this may explain some of the observed discrepancy in effect size. It must be noted, however, that this LD estimate is taken from CEU (Utah residents with Northern and Western European ancestry from the CEPH collection) HapMap samples as rs10830963 has not been genotyped in the GIH HapMap samples. In addition, imputation analyses in the Indian Asian study estimated that the strongest association signal for fasting glucose in this population was in fact the rs10830963 SNP [9], although this imputation was not able to utilise South Asian-specific LD patterns. It is interesting to note that a recent study of Indian Sikhs demonstrated that a low frequency variant (rs1374645) was associated with glucose levels, whereas rs10830963 was not [11]. Further studies of *MTNR1B* SNPs and their association with glucose levels in South Asians may be useful in fine-mapping the aetiological variant.

Variants in *TCF7L2* and *SLC30A8* have previously been associated with type 2 diabetes in our Punjabi populations [3], [6]. In addition to these variants, SNPs in *ADCY5* and *GLIS3* were associated with the disease in the current study. To our knowledge, this is the first time that either of these SNPs has been implicated in type 2 diabetes development in a South Asian population. The *ADCY5* gene encodes adenylate cyclase 5, an enzyme that catalyses the generation of cAMP, a second messenger vital in a number of biological processes. It has been demonstrated in a large meta-analysis of Europeans that the rs11708067 SNP within *ADCY5* is strongly associated (*p*≤3.6×10^−8^) with type 2 diabetes, fasting glucose levels and HOMA-B (a measure of β-cell function) but is not associated with HOMA-IR (a measure of insulin resistance) [1], [12]. This suggests that variants within this gene may exert their effect on disease risk through β-cell dysfunction and insulin secretion. In addition, a SNP within the *ADCY5* gene (rs9883204), in LD with the variant investigated in this study, is associated with foetal growth and birth weight [13]. Insulin is an important growth factor *in utero*, potentially providing a common mechanism linking reduced foetal growth with increased risk of type 2 diabetes. The *GLIS3* gene encodes the transcription factor GLIS family zinc finger 3 isoform, a protein that regulates target gene transcription and has been shown to play a key role in β-cell generation in mice [14], [15]. Rare functional mutations within the *GLIS3* gene lead to a syndrome of neonatal diabetes and congenital hyperthyroidism [16], and the rs7020673 SNP within the gene is robustly associated with type 1 diabetes [17]. The *GLIS3* rs7034200 SNP only displayed a weak association with type 2 diabetes in the MAGIC study [1], although there is evidence that this variant confers risk of the disease in a Chinese population [18]. As with the fasting glucose analyses, some potential disparity was apparent between our South Asian type 2 diabetes association results and those reported by the MAGIC study [1] (Figure 2), although the relatively small size of our cohort makes any differences difficult to quantify statistically. It is interesting to note that, excluding the *TCF7L2* SNP, three of the four variants most strongly associated with type 2 diabetes in the MAGIC study [1] (*MTNR1B* rs10830963, *PROX1* rs340874 and *GCKR* rs780094) have odds ratios of less than one in our South Asian populations (Figure 2).

The lack of association of many of the studied variants with fasting glucose and type 2 diabetes in our study cohort could be due to small sample size and low statistical power (Table 3). For the analysis of type 2 diabetes, this study had >80% power to detect the effect of just the *TCF7L2* SNP. The study was underpowered to detect the effect of any SNP on fasting glucose levels, assuming similar effect sizes to those seen in European populations (Table 3). Although the statistical evidence for heterogeneity is weak, it is also possible that the studied variants have different effect sizes in our Punjabi populations compared with Europeans. This may be due to differences in LD patterns between the two ethnic groups, as disease-associated SNPs derived from GWA studies are typically not aetiological variants. Previous findings that the *MTNR1B* and *G6PC2* variants genotyped in this study are associated, either directly or through imputation, with glucose levels in Indian Asians [9], however, make it unclear whether any potential differences in LD patterns are likely to contribute to our observed lack of association at these loci.

**Table 3:** Statistical power for fasting glucose and type 2 diabetes analyses in the combined UKADS/DGP study population.

				Type 2 diabetes analyses(n = 3262)		Fasting glucose analyses(n = 1163)	
Nearest gene	SNP	South Asian RAF	MAGIC RAF	MAGIC effect size(OR)	Power	MAGIC effect size(mmol/l)	Power
*MTNR1B*	rs10830963	0.40	0.30	1.09	0.48	0.067	0.75
*ADRA2A*	rs10885122	0.76	0.87	1.04	0.12	0.022	0.12
*C2CD4B*	rs11071657	0.68	0.63	1.03	0.09	0.008	0.06
*SLC30A8*	rs11558471	0.73	0.68	1.15	0.78	0.027	0.16
*CRY2*	rs11605924	0.49	0.49	1.04	0.14	0.015	0.09
*ADCY5*	rs11708067	0.76	0.78	1.12	0.57	0.027	0.15
*SLC2A2*	rs11920090	0.85	0.87	1.01	0.05	0.020	0.09
*FADS1*	rs174550	0.81	0.64	1.04	0.11	0.017	0.08
*GCK*	rs1799884^a^	0.15	0.16	1.07	0.20	0.062	0.43
*DGK/TMEM195*	rs2191349	0.60	0.52	1.06	0.25	0.030	0.22
*PROX1*	rs340874	0.59	0.52	1.07	0.32	0.013	0.08
*G6PC2*	rs560887^b^	0.84	0.70	0.97	0.08	0.075	0.60
*GLIS3*	rs7034200	0.48	0.49	1.03	0.10	0.018	0.11
*GCKR*	rs780094	0.73	0.62	1.06	0.21	0.029	0.18
*TCF7L2*	rs7903146^c^	0.32	0.31	1.40	1.00	0.023	0.14
*MADD*	rs7944584	0.78	0.75	1.01	0.05	0.021	0.11

RAF = risk (glucose-raising) allele frequency. Power was calculated using the South Asian RAF, the effect sizes reported in the MAGIC study [1], the sample sizes used in each analysis, an additive model and a significance level (α) of 0.05. For the type 2 diabetes analyses power calculations, a disease prevalence of 10% was assumed. For the fasting glucose analyses a population mean (SD) fasting glucose of 5.5 (0.6) mmol/l was used, as reported in Table 1.

a In the current study the *GCK* rs1799884 SNP was used as a proxy for the rs4607517 variant reported in MAGIC (r^2^ = 1.0 in CEU HapMap samples); the MAGIC RAF shown is for rs4607517.

b For the *G6PC2* rs560887 SNP, the glucose-raising allele reduces the risk of type 2 diabetes (with nominal significance) in the MAGIC study. In this instance, to calculate power for the type 2 diabetes analysis the minor allele frequency (0.16) and the inverse of the odds ratio (OR; 1.03) was used.

c The RAF for the *TCF7L2* rs7903146 SNP was not given in the MAGIC study; the RAF reported is for rs4506565 (r^2^ = 0.92 between the two variants in MAGIC).

In conclusion, our study of Punjabi populations demonstrated that 12 of 16 variants displayed β-values for fasting glucose with the same direction of effect as that seen in Europeans. In addition, we provide evidence that alleles of SNPs in *ADCY5* and *GLIS3* may confer risk of type 2 diabetes, the first time that this has been reported in South Asian populations.

## Supporting Information

Table S1 **Genotype distributions in the UKADS and DGP study populations.**
(DOCX)
