# Supplementary material for: Effects of 16 Genetic Variants on Fasting Glucose and Type 2 Diabetes in South Asians: ADCY5 and GLIS3 Variants May Predispose to Type 2 Diabetes
Source: PLoS One. 2011 Sep 20;6(9):e24710. doi: 10.1371/journal.pone.0024710 (PMC3176767; doi:10.1371/journal.pone.0024710)
Supplement: Table S1 — Genotype distributions in the UKADS and DGP study populations. (DOCX) [file pone.0024710.s001.docx]

|  |  |  | **UKADS** | | | | **DGP** | | | |
| --- | --- | --- | --- | --- | --- | --- | --- | --- | --- | --- |
|  |  |  | **Cases** | | **Controls** | | **Cases** | | **Controls** | |
| **Nearest gene** | **SNP** | **Allele (Risk/other)** | **RAF** | **Genotypes (0/1/2)** | **RAF** | **Genotypes (0/1/2)** | **RAF** | **Genotypes (0/1/2)** | **RAF** | **Genotypes (0/1/2)** |
| *MTNR1B* | rs10830963 | G/C | 0.40 | 320/386/145 | 0.42 | 139/201/71 | 0.39 | 311/367/138 | 0.39 | 444/513/200 |
| *ADRA2A* | rs10885122 | G/T | 0.78 | 45/275/517 | 0.79 | 17/135/255 | 0.77 | 51/262/477 | 0.75 | 72/435/636 |
| *C2CD4B* | rs11071657 | A/G | 0.68 | 82/362/385 | 0.70 | 34/178/196 | 0.66 | 105/331/355 | 0.68 | 130/468/540 |
| *SLC30A8* | rs11558471 | C/T | 0.73 | 69/317/451 | 0.71 | 43/153/211 | 0.77 | 50/267/468 | 0.74 | 85/420/634 |
| *CRY2* | rs11605924 | A/C | 0.50 | 201/407/208 | 0.48 | 109/204/89 | 0.48 | 229/362/197 | 0.49 | 300/560/285 |
| *ADCY5* | rs11708067 | A/G | 0.79 | 46/262/519 | 0.74 | 25/160/223 | 0.80 | 35/251/508 | 0.77 | 65/405/670 |
| *SLC2A2* | rs11920090 | T/A | 0.86 | 22/181/624 | 0.85 | 7/104/292 | 0.84 | 20/216/562 | 0.85 | 24/297/820 |
| *FADS1* | rs174550 | T/C | 0.81 | 31/248/554 | 0.81 | 15/123/269 | 0.84 | 22/207/559 | 0.81 | 40/344/752 |
| *GCK* | rs1799884 | A/G | 0.13 | 652/182/18 | 0.16 | 290/119/5 | 0.15 | 588/211/19 | 0.15 | 851/284/27 |
| *DGK/TMEM195* | rs2191349 | T/G | 0.60 | 130/404/298 | 0.62 | 69/165/165 | 0.63 | 123/341/336 | 0.60 | 193/536/417 |
| *PROX1* | rs340874 | C/T | 0.58 | 150/384/290 | 0.59 | 74/185/149 | 0.58 | 157/355/290 | 0.59 | 180/574/390 |
| *G6PC2* | rs560887 | C/T | 0.85 | 22/212/613 | 0.82 | 11/122/273 | 0.83 | 34/216/565 | 0.84 | 23/319/816 |
| *GLIS3* | rs7034200 | A/C | 0.51 | 200/411/218 | 0.48 | 110/205/92 | 0.53 | 185/369/231 | 0.48 | 303/544/268 |
| *GCKR* | rs780094 | C/T | 0.71 | 76/330/432 | 0.74 | 27/156/221 | 0.74 | 65/300/449 | 0.72 | 84/475/599 |
| *TCF7L2* | rs7903146 | T/C | 0.36 | 353/370/115 | 0.31 | 205/160/47 | 0.37 | 318/381/107 | 0.32 | 533/492/126 |
| *MADD* | rs7944584 | A/T | 0.78 | 49/274/509 | 0.77 | 24/141/248 | 0.80 | 39/249/518 | 0.78 | 56/387/684 |

Supplementary Table 1 – Genotype distributions in the UKADS and DGP study populations

UKADS = UK Asian Diabetes Study; DGP = Diabetes Genetics in Pakistan. Risk allele is the fasting glucose raising allele reported in Dupuis et al [1]; RAF = risk allele frequency. Genotypes are coded as 0,1 or 2 copies of the risk allele.

The *GCK* rs1799884 SNP was used as a proxy for the rs4607517 variant reported in Dupuis et al [1] (r^2^ = 1.0 in CEU HapMap samples).
